# Protective effect of omega-3 polyunsaturated fatty acids on sepsis via the AMPK/mTOR pathway

**DOI:** 10.1080/13880209.2023.2168018

**Published:** 2023-01-24

**Authors:** Peng Liu, Ming Li, Wei Wu, Anjie Liu, Honglin Hu, Qin Liu, Chengzhi Yi

**Affiliations:** a Wuhan Fourth Hospital, Wuhan, China; b Emergency Center, Zhongnan Hospital of Wuhan University, Wuhan, China

**Keywords:** Cecum ligation perforation, organ injury, inflammation

## Abstract

**Context:**

Sepsis is a systemic inflammatory response caused by infection, with high morbidity and mortality. Omega-3 polyunsaturated fatty acids (ω-3 PUFAs) have reported biological activities.

**Objective:**

This study explored the signaling pathways through which ω-3 PUFAs protect against sepsis-induced multiorgan failure.

**Materials and methods:**

Septic Sprague-Dawley (SD) rat model was established by the cecum ligation perforation (CLP) method. Rats were divided into control, sham, model, parenteral ω-3 PUFAs (0.5 g/kg) treatment, ω-3 PUFAs (0.5 g/kg) + AMPK inhibitor Compound C (30 mg/kg) treatment, and ω-3 PUFAs (0.5 g/kg) + mTOR activator MHY1485 (10 mg/kg) treatment groups. The serum inflammatory cytokines were measured using ELISA. Organ damage-related markers cTnI, CK, CK-MB, Cr, BUN, ALT, and AST were measured using an automated chemical analyzer. The AMPK/mTOR pathway in liver, kidney, and myocardial tissues was detected using western blot and qRT-PCR methods.

**Results:**

CLP treatment enhanced the secretion of pro-inflammatory cytokines and multi-organ related markers, along with increased p-AMPK/AMPK ratio (from 0.47 to 0.87) and decreased p-mTOR/mTOR ratio (from 0.33 to 0.12) in rats. The inflammation response and multi-organ injury induced by CLP treatment could be partially counteracted by 0.5 g/kg parenteral ω-3 PUFA treatment. The activated AMPK/mTOR pathway in CLP-induced rats was further promoted. Finally, Compound C and MHY1485 could reverse the effects of parenteral ω-3 PUFA treatment on sepsis rats.

**Discussion and conclusion:**

ω-3 PUFAs ameliorated sepsis development by activating the AMPK/mTOR pathway, serving as a potent therapeutic agent for sepsis. Further *in vivo* studies may validate potential clinical use.

## Introduction

Sepsis is a life-threatening and dysregulated host response caused by infections or infectious agents (Salomão et al. 2019). It is a common complication in patients with clinical trauma/burns and infections (Muñoz et al. 2019). Sepsis can also lead to septic shock and multiple organ dysfunction syndrome (MODS), which is the leading cause of death in critically ill patients, with a mortality rate between 30% and 70% (Ramírez 2013). Globally, there are an estimated 31.5 million cases of sepsis and 19.4 million cases of severe sepsis annually, with approximately 5.3 million deaths (Stephen et al. 2020). Despite the increasing understanding of the pathogenesis, the occurrence and exacerbation of sepsis remains unclear, with limited treatment efficacy.

Omega-3 polyunsaturated fatty acids (ω-3 PUFAs), including eicosapentaenoic acid (EPA) and docosahexaenoic acid (DHA), are mainly found in marine organisms and their products (Shahidi and Ambigaipalan 2018). ω-3 PUFAs help provide energy and nutrients to the body, along with performing anti-thrombotic function, regulating immunity, lipid metabolism, and gene expression (Gutiérrez et al. 2019; Wang et al. 2019; Daak et al. 2020). Meanwhile, ω-3 PUFAs may play a role in suppressing inflammation, which in turn helps control infection and improve outcomes (Pradelli et al. 2020; Weill et al. 2020). The therapeutic effects of ω-3 PUFAs in sepsis have recently gained attention (Chen et al. 2018). However, the pathways through which ω-3 PUFAs regulate sepsis remain unclear.

The cecum ligation perforation (CLP) model is commonly used for sepsis modeling as it is easy to manipulate, is reproducible, and similar to the progression of human sepsis (Deng et al. 2018; Liang et al. 2019). Notably, the CLP model further enhances the clinical validity of the model by reconstructing the hemodynamic and metabolic alteration patterns of early human sepsis, and by mimicking the course of human disease in terms of the regulatory processes of specific cell types and host immune response patterns (Farrag et al. 2015; Olivier et al. 2017). Another advantage of the CLP model is that it can be designed for different severity levels of sepsis depending on the length of the ligated cecum, number of perforations, and diameter of the puncture needle (Song et al. 2016). Thus, in this study, a rat sepsis model is developed according to the CLP method. After constructing the CLP model, the expressions and roles of ω-3 PUFAs in regulating inflammation response and the AMPK/mTOR pathway are evaluated.

This study explores the effects and mechanisms of ω-3 PUFAs on sepsis-induced multiorgan failure.

## Materials and methods

### Construction of CLP-induced septic rats

60 SPF-grade Sprague-Dawley (SD) rats (female, 7–8 weeks old, weighing approximately 200–220 g) were purchased from Charles River Laboratories, Inc. The rats were housed in a temperature-controlled room (22–24 °C) and humidity (60–65%) with a 12 h light/dark cycle with free access to food and water. The disposal of animals during the experiments was in accordance with relevant animal ethics standards. The current study was approved by the Committee on the Use and Care of Animals of the Wuhan Myhalic Biotechnology Co., Ltd. (Approval number: 2020-042501).

The septic rat model was prepared using the CLP method. SD rats were under aseptic rearing for over 1 week with free access to water and food. After ether-inhalation anesthesia, the toes of the rats were clamped using hemostatic forceps, and sutures were wrapped around the rats’ incisors. The abdomen was routinely disinfected with iodophor, and a longitudinal incision, approximately 1.5 cm in length, was made in the midline of the abdomen. Then, the abdominal cavity was carefully opened, and the mesentery and cecum were freed with toothless forceps, leaving the small and large intestines in the abdominal cavity. From the distal apex of the cecum as the starting point, line 4 was tied at the midpoint of the cecum, and a single penetration of the cecum was undertaken at the mesenteric end to the opposite side using a 21-gauge needle. The peritoneum, muscle tissue, and skin were closed in layers with sutures. The entire experiment was conducted at 22–25 °C, and the rats were resuscitated by subcutaneous injection of saline (3 mL/100 g). Immediately after the operation, the rats were put back into the cage and fed in separate cages to resume regular diet and water supplementation.

Rats were randomly divided into 6 groups (*n* = 10): Control; Sham; Model; Model + ω-3 PUFAs; Model + ω-3 PUFAs + Compound C; and Model + ω-3 PUFAs + MHY1485. The sham group only did open and separate the distal end of the cecum with closed abdominal surgery. An hour after the CLP was established, parenteral ω-3 PUFA solution (0.5 g/kg; Omegaven^®^, Fresenius Kabi, Germany), AMPK inhibitor Compound C (30 mg/kg; Biovision, San Francisco, CA), and mTOR agonist MHY1485 (10 mg/kg; MedChemExpress, Shanghai, China) were intraperitoneally injected into rats to construct different treatment groups. In the model group, an equal amount of saline was injected into the tail vein (Jiang et al. 2019).

In this experiment, the animals were sacrificed by cervical dislocation after anesthesia 24 h after surgery, and blood was collected from the heart. Liver, kidney, and myocardial tissues were excised and collected for subsequent experiments.

### ELISA

Blood sample (4 mL) was collected from each rat heart before execution and allowed to clot naturally for 10 min. After centrifugation at 1000 *g* at 4 °C for 20 min, the levels of IL-1β (Rat IL-1β ELISA Kit: cat. no. PI303), TNF-α (Rat TNF-α ELISA Kit: cat. no. PT516), IL-6 (Rat IL-6 ELISA Kit: cat. no. PI328), IL-10 (Rat IL-10 ELISA Kit: cat. no. PI525), IFN-γ (Rat IFN-γ ELISA Kit: cat. no. PI510), and IL-17 (Rat IL-17 ELISA Kit: cat. no. PI548) in the serum of rats were measured by ELISA using the corresponding commercial kits (Beyotime, Shanghai, China). The operation was conducted strictly according to the manufacturer’s instructions. The standards were diluted according to the multiplicative dilution method, and a standard curve was prepared for each assay. Three replicate wells were set up for each sample, and each experiment was repeated three times.

### Measurement of serological parameters

Serological parameters cardiac troponin I (cTnI), creatine kinase (CK), creatine kinase-MB (CK-MB), creatinine (Cr), blood urea nitrogen (BUN), alanine transaminase (ALT), and aspartate transaminase (AST) were measured using biochemical estimation kits (Human diagnostics) on an automatic chemistry analyzer (Beckman Coulter, USA) as per the manufacturer’s instructions.

### qRT-PCR analysis

The TRIzol reagent was applied to extract RNA from septic rat organ tissues (liver, kidney, and cardiac tissues). Then, the concentrations of RNA were measured using the MaestroNano Pro spectrophotometer. Thereafter, cDNA was synthesized from RNA using the Bestar qPCR RT kit, and the PCR reaction was conducted using SYBR Green qPCR Master Mix, according to the manufacturer’s instructions. The PCR amplification conditions were as follows: initial denaturation at 95 °C for 5 min, 40 cycles of 95 °C for 10 s, 55 °C for 30 s, and 72 °C for 20 s. The ABI 7500 system was utilized for the measurements, using the 2^-△△CT^ method. The primer sequences were as follows: AMPK forward, 3′-CACCCTGAAAGAGTACCGT-5′; AMPK reverse, 3′-CATTTTGCCTTCCGTACACCT-5′; mTOR forward, 5′-AGCAGAGAAAGGTTTTGATG-3′; mTOR reverse, 5′-GATCTCCTCCATCTCTTCTC-3′; GAPDH forward, 5′-ACAGTTGCATGTAGACT-3′; GAPDH reverse, 5′-TTTTTGGTTGAGCACAGG-3′.

### Western blot assay

Radio Immunoprecipitation Assay (RIPA) solution with PMSF (1 mM) was used to isolate proteins from CLP-induced septic rat liver, kidney, and cardiac tissues. The initial protein concentrations were measured using the BCA protein assay kit. After the separation of proteins using 10% SDS-PAGE, PVDF membranes were used to transfer proteins electronically. After sealing in 5% BSA for 2 h, the membranes were incubated overnight at 4 °C with primary antibodies against p-AMPK (1:1000; ab133448; Abcam, Shanghai, China), AMPK (1:1000; ab207442; Abcam, Shanghai, China), p-mTOR (1:1000; ab109268; Abcam, Shanghai, China), mTOR (1:1000; ab32028; Abcam, Shanghai, China), and GAPDH (1:2500; ab9485; Abcam, Shanghai, China). The membranes were washed in triplicate with PBST and then the incubation was continued for 2 h using HRP-conjugated goat anti-rabbit IgG (1:1000; ab181662; Abcam, Shanghai, China) as the secondary antibody. The protein bands were visualized using the ECL detection reagent (Beyotime Institute of Biotechnology). Finally, protein concentrations were quantified using the ImageJ software.

### Statistical analysis

Data in this study were analyzed using the SPSS 23.0 software. One-way analysis of variance (ANOVA) followed by Tukey’s *post-hoc* test was used to analyze the differences between groups. All data were expressed as mean ± standard deviation (SD). Statistical significance was set at *p* < 0.05.

## Results

### ω-3 PUFAs treatment ameliorated CLP-induced inflammation response in septic rats

**Figure 1.**
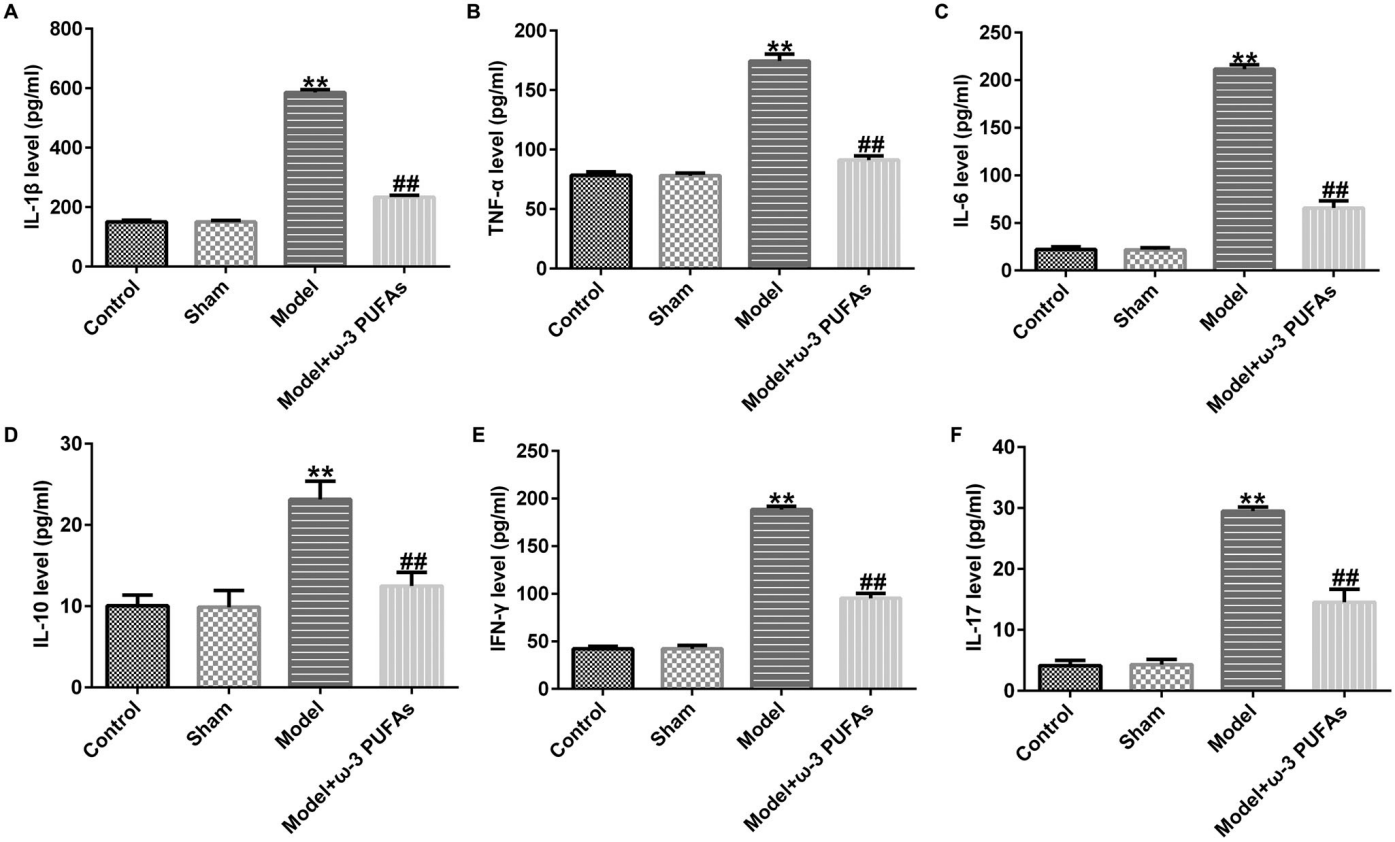
Secretion of inflammatory cytokines in blood samples from rats administered with CLP. (A) IL-1β; (B) TNF-α; (C) IL-6; (D) IL-10; (E) IFN-γ; (F) IL-17. **p < 0.01 vs. Sham; ^##^*p* < 0.01 vs. Model.

After CLP treatment, blood samples were collected from rats in different groups. The inflammation response was evaluated against inflammatory cytokines using ELISA. As shown in Figure 1(A–F), the expressions of IL-1β, TNF-α, IL-6, IL-10, IFN-γ, and IL-17 were significantly increased in the model group as compared to those in the sham group, whereas there was no difference between the sham and control groups. Additionally, parenteral ω-3 PUFA treatment partially restored the increase in IL-1β, TNF-α, IL-6, IL-10, IFN-γ, and IL-17 induced by the CLP treatment.

### ω-3 PUFAs treatment diminished multi-organ injury induced by CLP treatment

**Figure 2.**
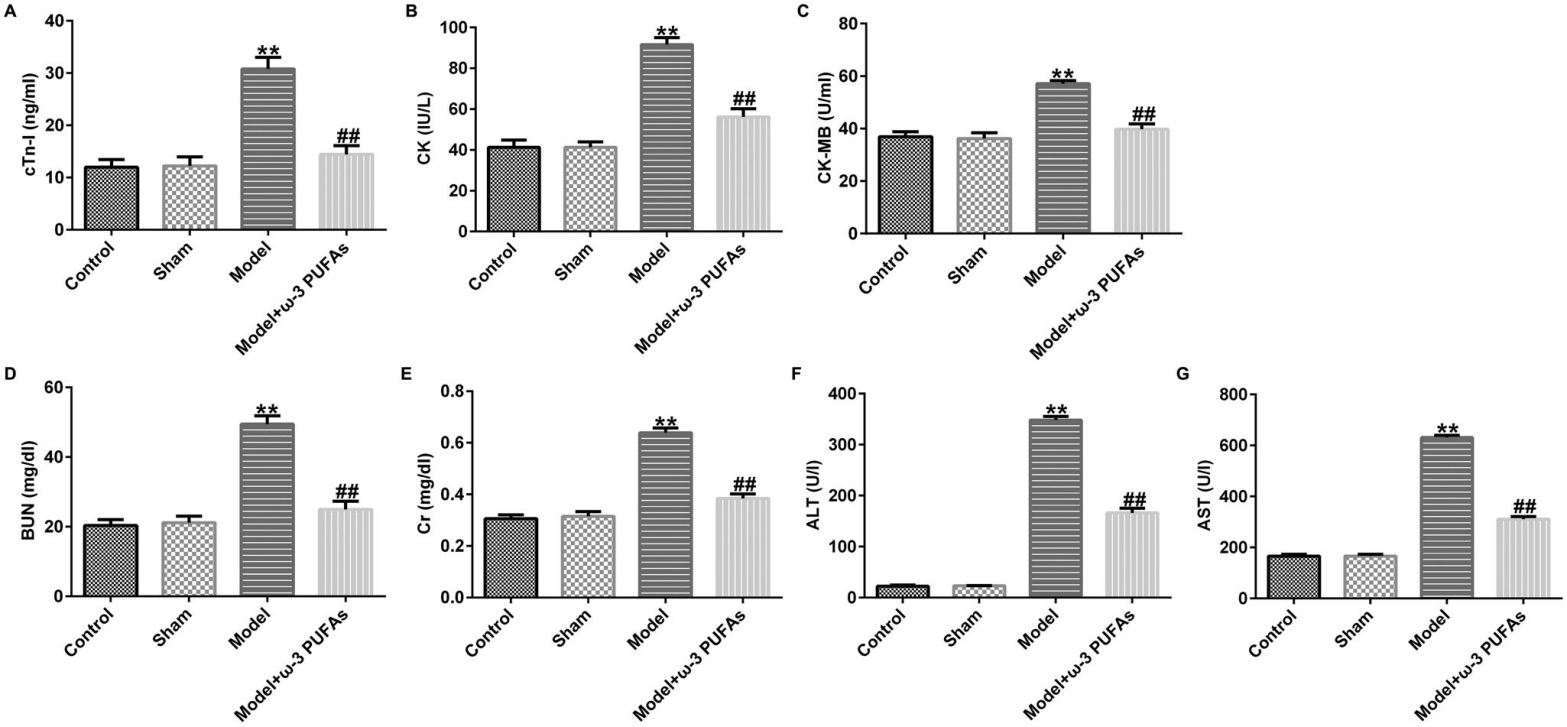
Levels of organ damage-related markers in rats after LPS challenge. (A) cTnI; (B) CK; (C) CK-MB; (D) BUN; (E) Cr; (F) ALT; (G) AST. CTnI, cardiac troponin I; CK, creatine kinase; CK-MB, creatine kinase-MB; Cr, creatinine; BUN, blood urea nitrogen; ALT, alanine transaminase; AST, aspartate transaminase. **p < 0.01 vs. Sham; ^##^*p* < 0.01 vs. Model.

In order to verify the effect of ω-3 PUFAs on multi-organ injury induced by CLP surgery, we measured cardiac, kidney, and liver injury indicators in different groups. As depicted in Figure 2(A–G), after CLP treatment, the secretions of cardiac parameters (cTnI, CK, and CK-MB) and kidney and liver injury markers (Cr, BUN, ALT, and AST) were prominently augmented as compared to those in the sham and control groups. However, the augment in the model group could be reversed after treatment with parenteral ω-3 PUFA solutions.

### ω-3 PUFAs activated AMPK/mTOR pathway in sepsis

**Figure 3.**
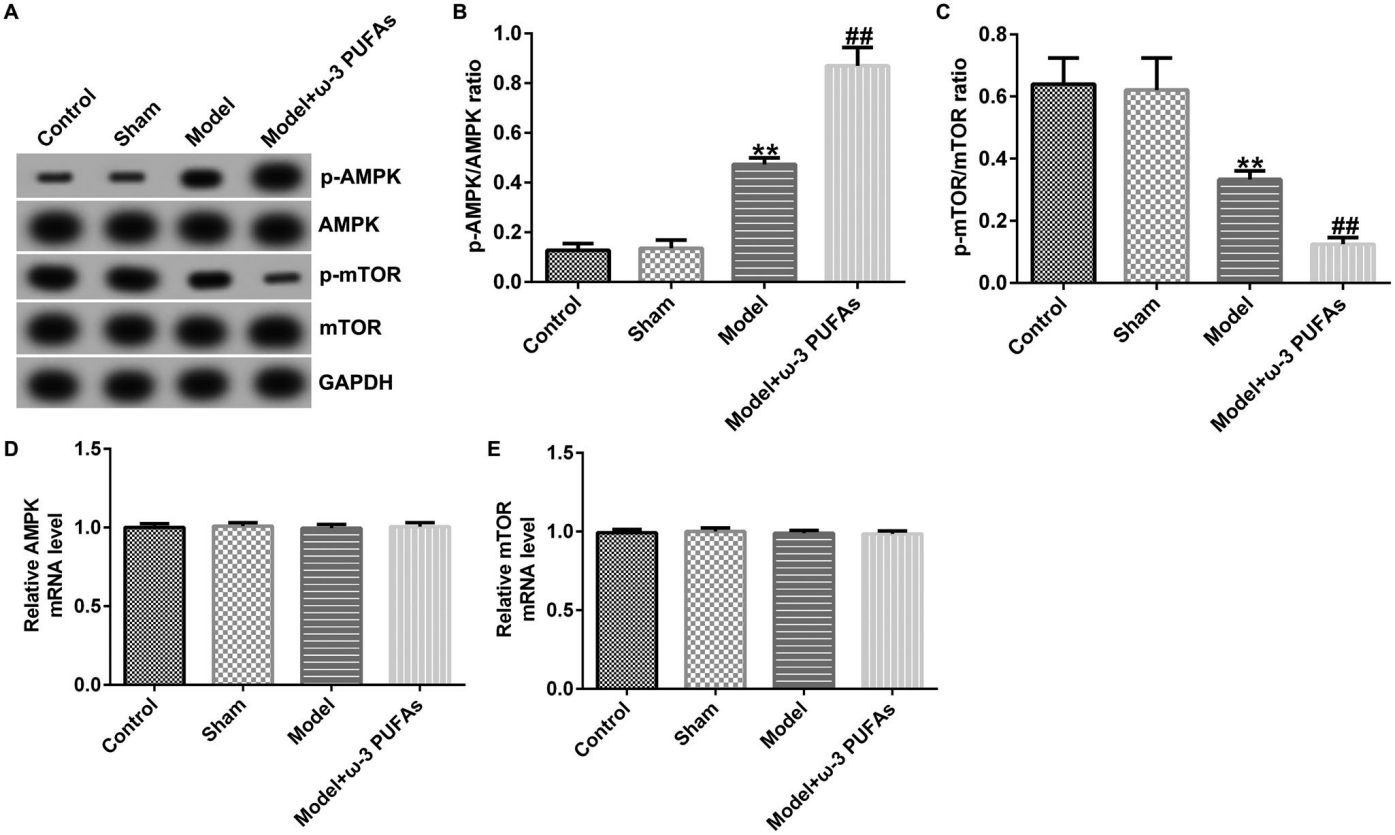
AMPK/mTOR pathway in cardiac muscle tissues from CLP-treated rats. (A) Protein expressions of AMPK, p-AMPK, mTOR and p-mTOR. (B) Quantified results of p-AMPK. (C) Quantified results of p-mTOR. (D and E) mRNA levels of AMPK and mTOR evaluated using qRT-PCR. **p < 0.01 vs. Sham; ^##^*p* < 0.01 vs. Model.

**Figure 4.**
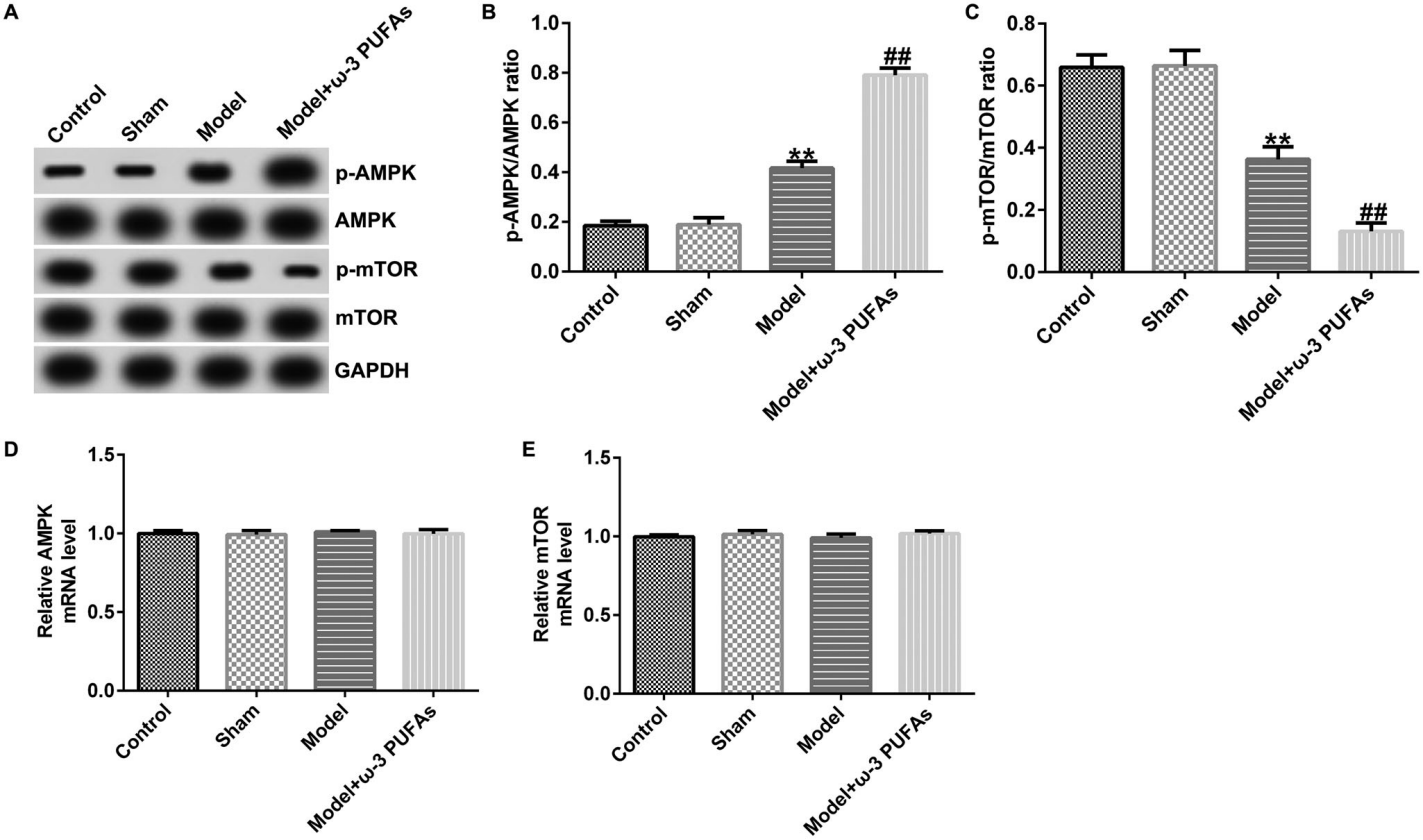
AMPK/mTOR pathway in kidney tissues from CLP-treated rats. (A) Protein expression of AMPK, p-AMPK, mTOR, and p-mTOR. (B) Quantified results of p-AMPK. (C) Quantified results of p-mTOR. (D and E) mRNA levels of AMPK and mTOR evaluated using qRT-PCR. **p < 0.01 vs. Sham; ^##^*p* < 0.01 vs. Model.

**Figure 5.**
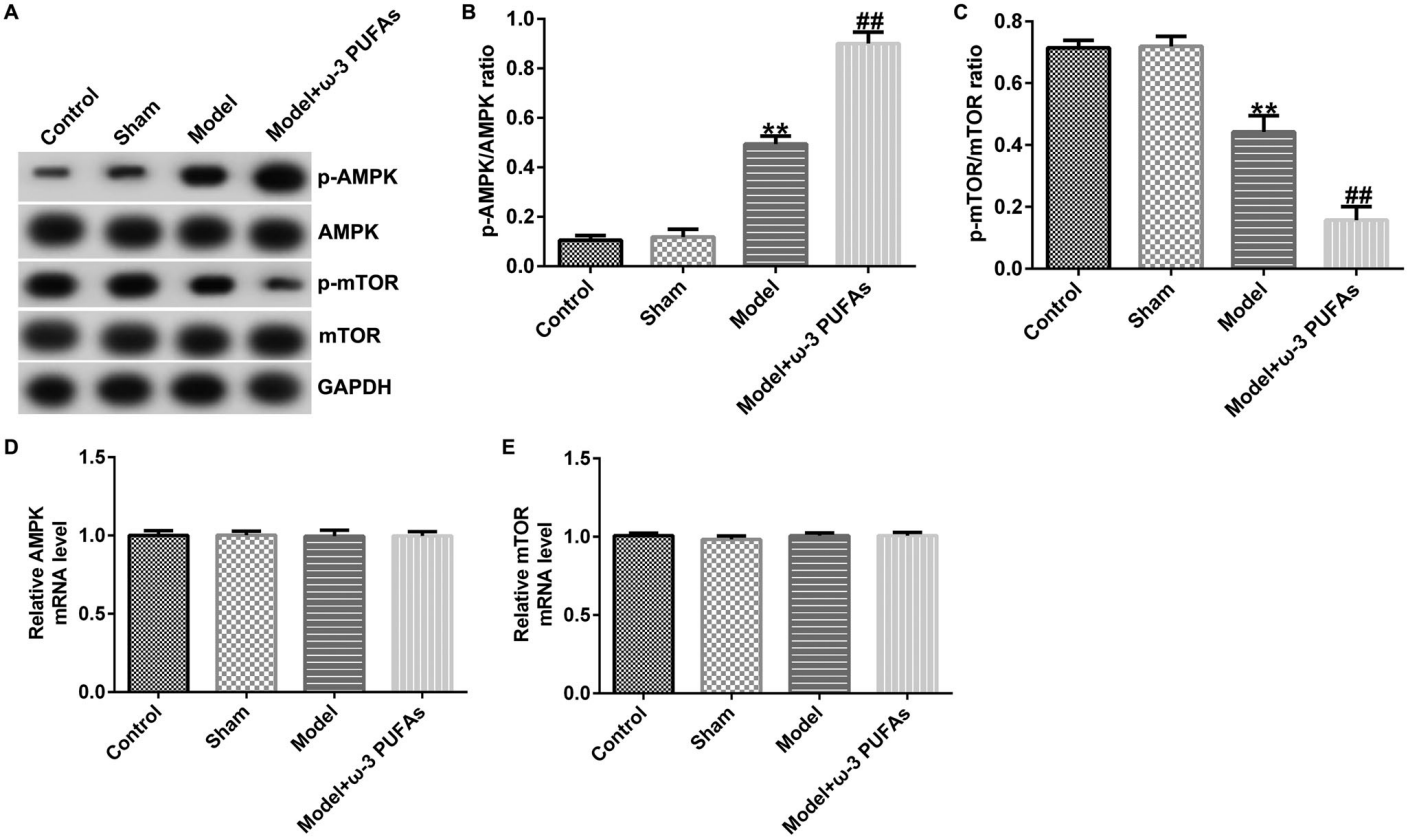
AMPK/mTOR pathway in liver tissues from CLP-treated rats. (A) Protein expression of AMPK, p-AMPK, mTOR and p-mTOR. (B) Quantified results of p-AMPK. (C) Quantified results of p-mTOR. (D and E) mRNA levels of AMPK and mTOR evaluated using qRT-PCR. **p < 0.01 vs. Sham; ^##^*p* < 0.01 vs. Model.

To confirm whether the AMPK/mTOR pathway was involved in sepsis after parenteral ω-3 PUFA treatment, the protein expressions of p-AMPK and p-mTOR were determined using the western blot assay. As demonstrated in Figures 3–5, CLP treatment could enhance p-AMPK protein expression while hindering p-mTOR level, along with increased p-AMPK/AMPK ratio and diminished p-mTOR/mTOR ratio. Compared with the model group, parenteral ω-3 PUFAs enhanced the effects of CLP treatment on p-AMPK and p-mTOR protein expression. There were no significant differences in AMPK and mTOR mRNA expressions.

### ω-3 PUFAs improved inflammation response in septic rats after experiencing CLP treatment through regulating the AMPK/mTOR pathway

**Figure 6.**
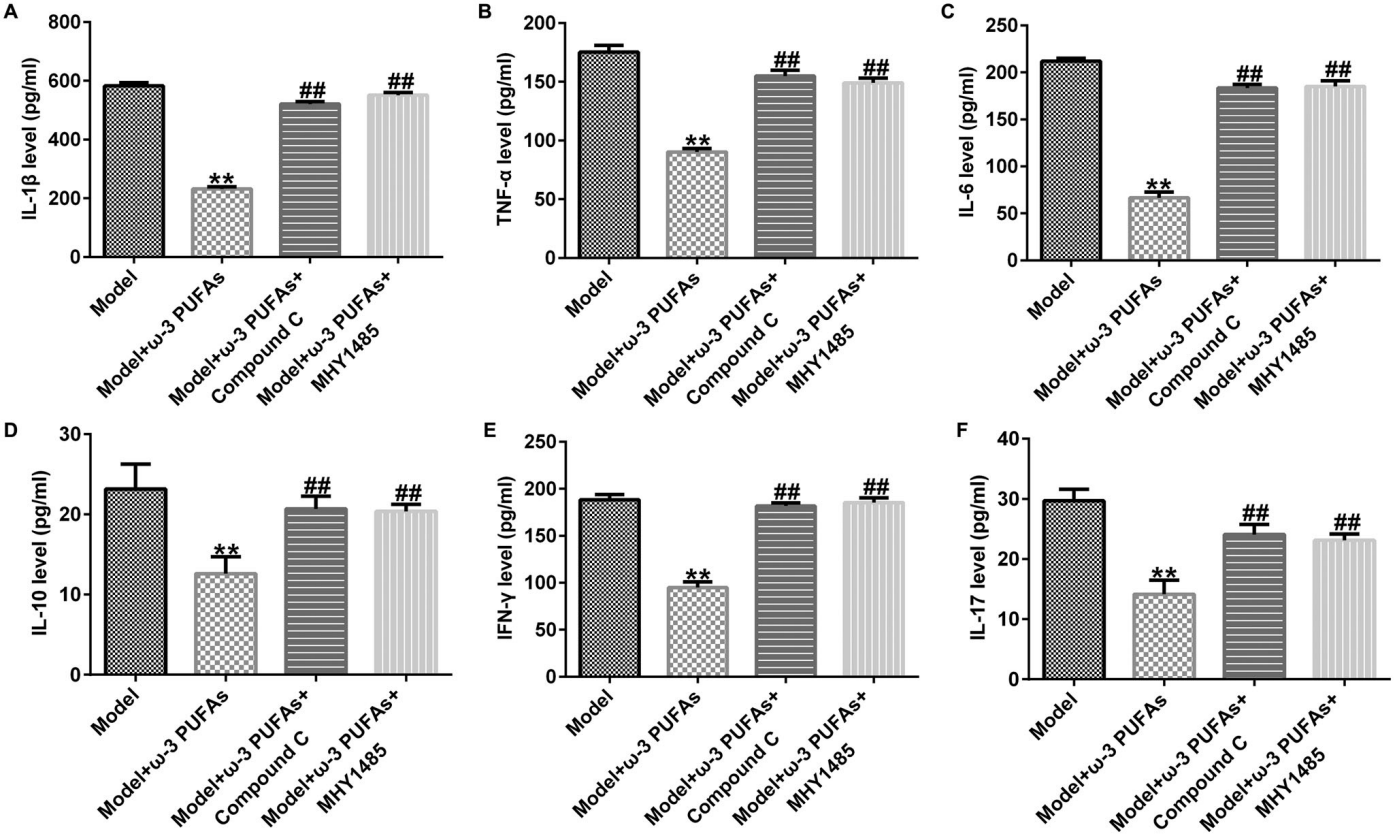
Secretion of inflammatory cytokines in blood samples from rats after treatment. (A) IL-1β; (B) TNF-α; (C) IL-6; (D) IL-10; (E) IFN-γ; (F) IL-17. **p < 0.01 vs. Model; ^##^*p* < 0.01 vs. Model + ω-3 PUFAs.

In order to verify whether the AMPK/mTOR pathway was activated in sepsis-induced inflammation, we injected AMPK inhibitor Compound C or mTOR agonist MHY1485 into septic rats. As shown in Figure 6, parenteral ω-3 PUFA treatment could mitigate inflammation response induced by the CLP treatment in rats with sepsis. However, the mitigation could be restored by injecting Compound C or MHY1485, suggesting that the AMPK/mTOR pathway was activated in regulating inflammation response during sepsis.

### ω-3 PUFAs mitigated septic rat cardiac, kidney and liver injury under CLP challenge via the AMPK/mTOR pathway

**Figure 7.**
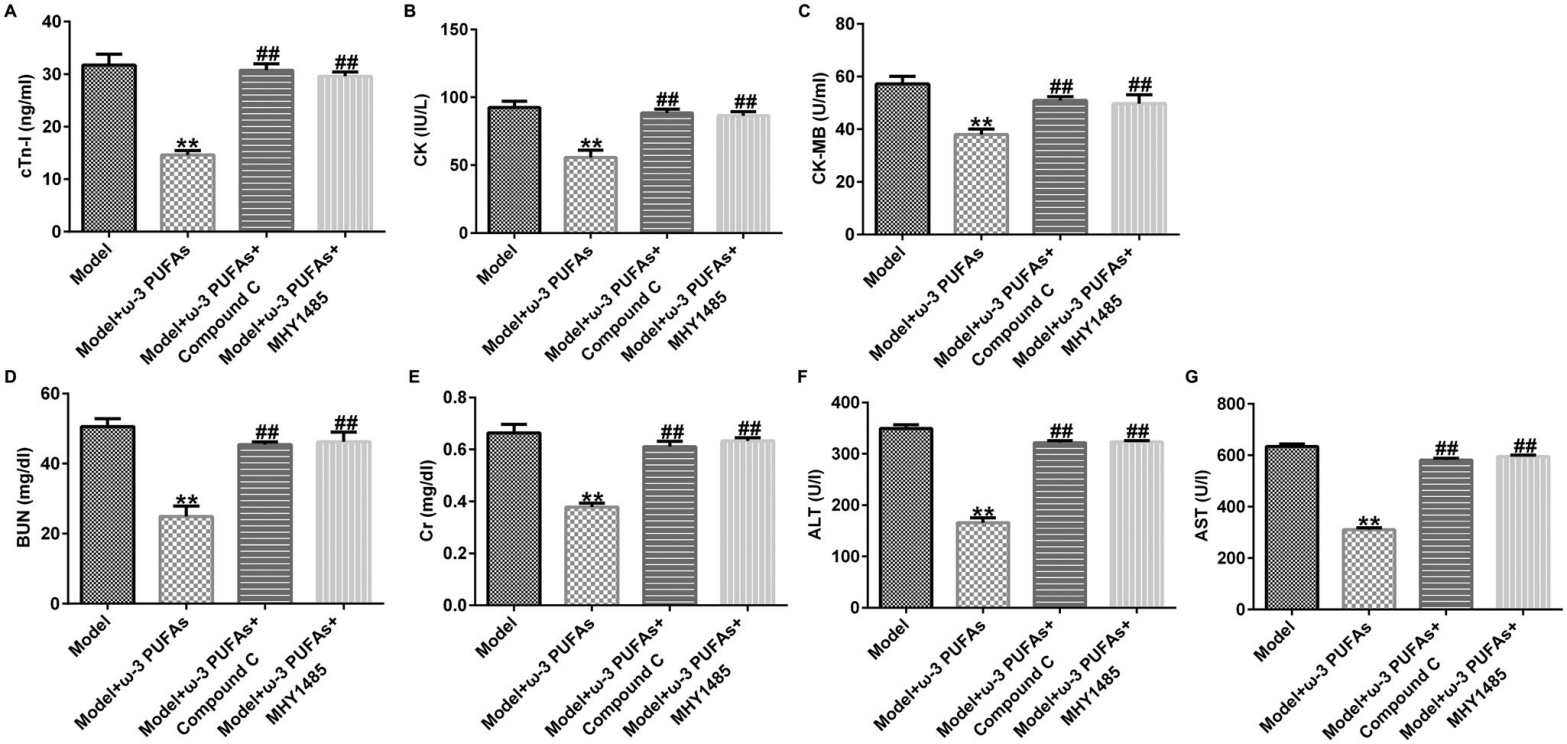
Levels of organ damage-related markers in rats with sepsis after treatment. (A) cTnI; (B) CK; (C) CK-MB; (D) Cr; (E) BUN; (F) ALT; (G) AST. CTnI, cardiac troponin I; CK, creatine kinase; CK-MB, creatine kinase-MB; Cr, creatinine; BUN, blood urea nitrogen; ALT, alanine transaminase; AST, aspartate transaminase. **p < 0.01 vs. Model; ^##^*p* < 0.01 vs. Model + ω-3 PUFAs.

As shown in Figure 7, we found that parenteral ω-3 PUFA treatment could ameliorate septic rat cardiac, kidney, and liver injury after CLP treatment by reducing the secretion of cTnI, CK, CK-MB, Cr, BUN, ALT, and AST. Additionally, compared with the model + ω-3 PUFAs group, injection with Compound C or MHY1485 could partially restore the reduction in cTnI, CK, CK-MB, Cr, BUN, ALT, and AST levels (Figure 7).

### ω-3 PUFAs protected against sepsis in CLP-induced rats by directly activating the AMPK/mTOR pathway

**Figure 8.**
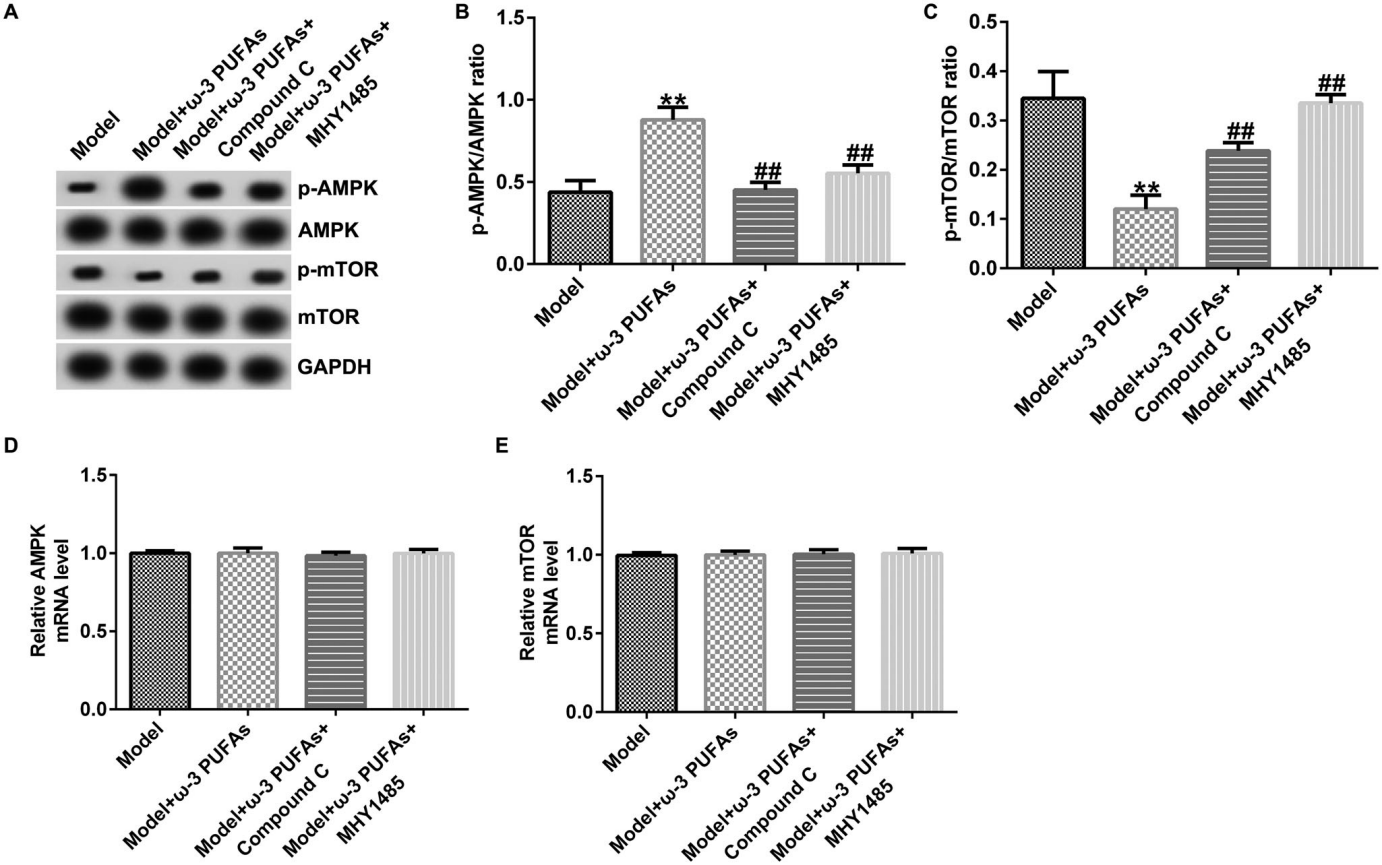
AMPK/mTOR pathway in cardiac muscle tissues from CLP-treated rats. (A) Protein expression of AMPK, p-AMPK, mTOR and p-mTOR. (B) Quantified results of p-AMPK. (C) Quantified results of p-mTOR. (D and E) mRNA levels of AMPK and mTOR evaluated using qRT-PCR. **p < 0.01 vs. Model; ^##^*p* < 0.01 vs. Model + ω-3 PUFAs.

**Figure 9.**
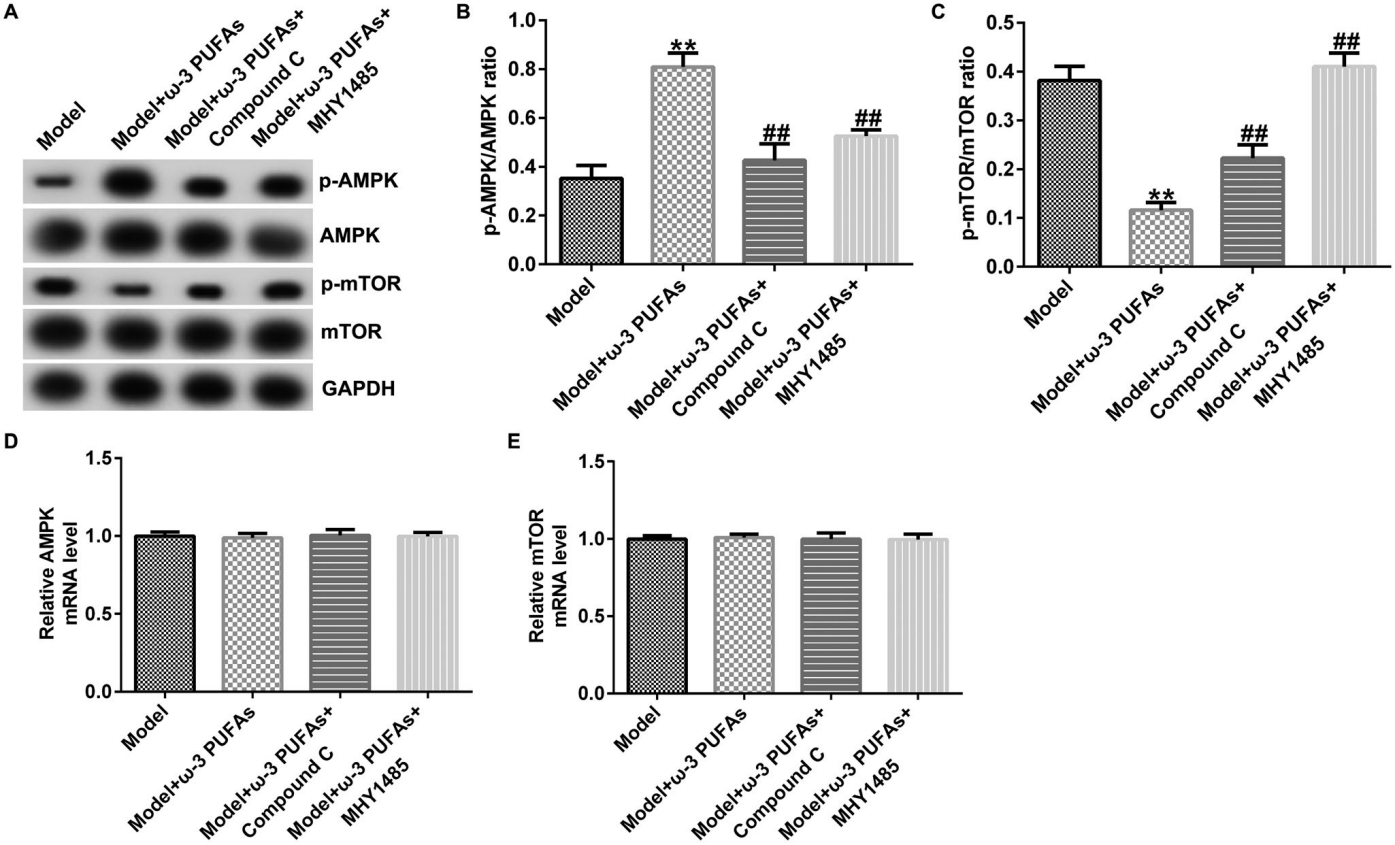
AMPK/mTOR pathway in kidney tissues from CLP-treated rats. (A) Protein expression of AMPK, p-AMPK, mTOR and p-mTOR. (B) Quantified results of p-AMPK. (C) Quantified results of p-mTOR. (D and E) mRNA levels of AMPK and mTOR evaluated using qRT-PCR. **p < 0.01 vs. Model; ^##^*p* < 0.01 vs. Model + ω-3 PUFAs.

**Figure 10.**
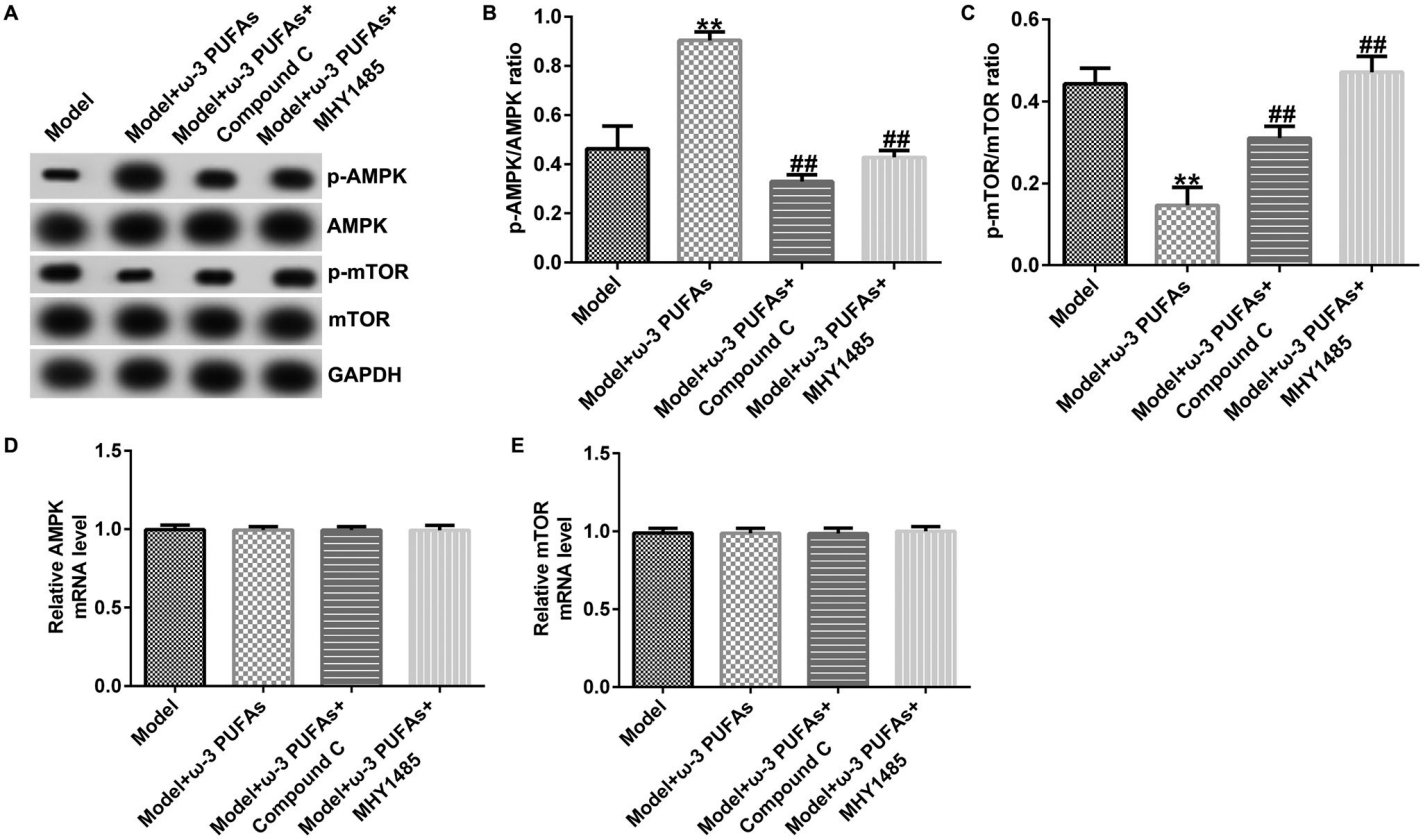
AMPK/mTOR pathway in liver tissues from CLP-treated rats. (A) Protein expression of AMPK, p-AMPK, mTOR, and p-mTOR. (B) Quantified results of p-AMPK. (C) Quantified results of p-mTOR. (D and E) mRNA levels of AMPK and mTOR evaluated using qRT-PCR. **p < 0.01 vs. Model; ^##^*p* < 0.01 vs. Model + ω-3 PUFAs.

The participation of the AMPK/mTOR pathway in regulating sepsis-induced multi-organ injury after parenteral ω-3 PUFA treatment was confirmed by the western blot and qRT-PCR analyses. As shown in Figures 8–10, there were no significant differences in AMPK and mTOR mRNA expression among different groups. Nevertheless, compared with the model group, parenteral ω-3 PUFAs treatment could increase p-AMPK expression and p-AMPK/AMPK ratio while inhibiting the p-mTOR level and p-mTOR/mTOR ratio (Figures 8–10). Meanwhile, the variance in p-AMPK and p-AMPK could be partially reversed by injecting Compound C or MHY1485.

## Discussion

Sepsis is a common and frequent disease in the field of critical care medicine and has become a severe public health burden (Corl et al. 2019). The ideal time to observe multi-organ injury in rats with sepsis is 24 h after modeling (Mertens et al. 2020; Zhang WQ et al. 2021). Therefore, the observation endpoint was set at 24 h after CLP treatment in this experiment. We found that the CLP treatment prominently increased cardiac, kidney, and liver injury markers and pro-inflammatory cytokines, which verified that the CLP model was successfully constructed.

Previous studies have demonstrated the significant regulatory effects of ω-3 PUFAs on inflammation, and these have recently become a research hotspot for dietary intervention and treatment of several chronic diseases. For instance, Galán-Arriero et al. (2017) showed that ω-3 PUFAs could reduce the occurrence of neuroinflammation, counteract oxidative stress, decrease the rate of apoptosis, and diminish neurological damage. In a model of hypoxic-ischemic brain injury, early administration of ω-3 PUFAs reduced the number of damaged cells and neuronal swelling (Zhang WT et al. 2016). Another study elucidated that perinatal supplementation with ω-3 PUFAs could ameliorate neonatal hypoxic/ischemic brain injury by promoting phosphatidylserine formation, thereby inhibiting neuronal cell death (Zhang WT, Liu, et al. 2015). Wang et al. (2020) illustrated that supplementation with ω-3 PUFAs could reduce mortality in patients with sepsis, along with shortening the duration spent in ICU. Tian et al. (2016) revealed the anti-inflammatory effects of ω-3 PUFAs on CLP-induced sepsis, which further improved survival rates. The interaction of pro-inflammatory and anti-inflammatory factors is the key to the development of sepsis (Venet and Monneret 2018). Additionally, the onset of sepsis promotes the release of a large number of cytokines, which can trigger a series of inflammatory responses, leading to organ dysfunction (Rocha et al. 2021). In our study, we found that injecting parenteral ω-3 PUFA solutions reduced IL-1β, TNF-α, IL-6, IL-10, IFN-γ, and IL-17 levels and hindered the secretion of multi-organ injury markers induced by CLP treatment, indicating the protective role of ω-3 PUFAs in sepsis.

To explore the effects of ω-3 PUFA solutions on multi-organ injury induced by CLP treatment (Greco et al. 2017), we examined markers of myocardial injury (CTn-I, CK, and CK-MB), kidney and liver injury markers (Cr, BUN, ALT, and AST) secretion (Liu et al. 2017; Chen et al. 2021). The findings of current study indicated that the secretion of cTnI, CK, CK-MB, Cr, BUN, ALT, and AST induced by CLP treatment could be reversed by parenteral ω-3 PUFAs solutions treatment, suggesting the protective role of ω-3 PUFA solutions in sepsis induced multi-organ injury.

The AMPK pathway is activated when AMP and ADP cellular levels are elevated due to various physiological stresses or pharmacological inducers (Carling 2017). Once activated, AMPK regulates protein, lipid, and sugar metabolism as well as autophagy and mitochondrial homeostasis, covering almost the entire physiological and metabolic activities of living organisms (Fullerton 2016; Casanova et al. 2019). During conditions of nutrient deficiency, AMPK is a metabolic checkpoint that inhibits cell growth (Zhang FJ, Zhang, et al. 2015; Jellusova 2020). This mechanism is achieved by inhibition of the mTORC1 pathway (Xu et al. 2019). AMPK can directly block the ability of the mTOR kinase complex to phosphorylate its substrates (Inoki et al. 2012). The mTOR signaling pathway, as a central coordinator of cell growth, is closely related to the development of liver fibrosis (Wang et al. 2019). In sepsis, upregulated p-AMPK and downregulated p-mTOR were observed in the CLP model (Zhang J et al. 2017; Zhao et al. 2018; Liu, Li, et al. 2020; Liu, Xu, et al. 2020). Meanwhile, DHA, a component of ω-3 PUFAs, was found to regulate the AMPK/mTOR signaling pathway in cancer cells (Jing et al. 2011). In the present study, we injected parenteral ω-3 PUFA solutions along with AMPK inhibitor Compound C and mTOR agonist MHY1485 into CLP-induced rats with sepsis. The results indicated that Compound C or MHY1485 could partially reverse the ameliorated inflammation response and multi-organ injury induced by ω-3 PUFAs in sepsis, indicating that ω-3 PUFAs protected against sepsis by directly regulating the AMPK/mTOR pathway.

## Conclusions

This research indicated that ω-3 PUFAs could alleviate CLP-induced inflammatory response and multi-organ damage in rats with sepsis by regulating the AMPK/mTOR signaling pathway; thus, may be used as a potential therapeutic agent for the treatment of sepsis. We will further explore the effect and specific mechanism of the components of omega-3 PUFAs in the animal model of sepsis in our next research.

## Data Availability

The datasets used and/or analyzed during the current study are available from the corresponding author upon reasonable request.
